# Computer-assisted teaching of bilateral sagittal split osteotomy: Learning curve for condylar positioning

**DOI:** 10.1371/journal.pone.0196136

**Published:** 2018-04-25

**Authors:** Charles Savoldelli, Emmanuel Chamorey, Georges Bettega

**Affiliations:** 1 Department of Oral and Maxillofacial Surgery, Head and Neck Institute, University Hospital of Nice, Nice, France; 2 Biostatistic Department, Centre Antoine Lacassagne, Nice, France; 3 Department of Oral and Maxillofacial surgery, Centre Hospitalier Annecy-Genevois, Metz-Tessy, France; 4 Plastic and Maxillofacial Surgery Department, Grenoble University Hospital, BP, Grenoble, France; Virginia Commonwealth University, UNITED STATES

## Abstract

Bilateral sagittal split osteotomy (BSSO) is a widely-performed procedure in orthognathic surgery for the correction of dentofacial deformity. Condylar positioning is a critical step during BSSO to maximize functional and morphological results. The unsuitable positioning of condyles represents one of the causative mechanisms that may induce temporomandibular joint noxious effects after BSSO. Repositioning devices can assist surgeons in maintaining the preoperative condylar position; however, empirical repositioning methods based on experience gained are still commonly used. Trainee learning curves are difficult to assess. The aim of this study was to evaluate the relevance of computer-assisted surgery in the acquisition of condylar positioning skills. Forty-eight patients underwent BSSO performed by six maxillofacial trainees (four junior residents and two senior experienced residents). A condyle positioning system (CPS) was used by a senior surgeon to record a condylar position score during the procedure. Firstly, scores were recorded when the trainee manually positioned the condyle without access to the CPS score (phase 1) and then when the trainee positioned the condyle and performed osteosynthesis with visual access to the CPS score (phase 2). Six parameters describing condylar three-dimensional motions were assessed: translational motion from top to bottom (TB), back to front (BF), and left to right (LR), axial rotation (AR), sagittal rotation (SR), frontal rotation (FR), and a total score (TS). There were no significant differences between junior and senior residents in condyle positioning without access to the CPS. Condyles were significantly better positioned during phase 2 with access to the CPS (*p*<0.001). Over time, use of the CPS (phase 2) produced significantly quicker improvements in scores (*p* = 0.042). For those teaching surgeries to trainees, computer-assisted devices can potentially result in more rapid learning curves than traditional “observations-imitation” models. Use of a CPS by trainees facilitated condylar repositioning that resulted in an accurate occlusal result and avoidance of adverse effects on the temporomandibular joint.

## Introduction

Bilateral sagittal split osteotomy (BSSO) is a widely-performed orthognathic procedure for the correction of dentofacial deformity. Maintaining the condylar position during BSSO remains technically difficult and is related to the surgeon’s experience. Most maxillofacial surgeons use an intuitive repositioning technique consisting of manual placement of the condylar process in the superior and posterior glenoid fossa [1,2]. Centric condyle relation is considered as a reference positioning and used for reproducibility. Acquisition of condyle repositioning skills is critical in achieving BSSO competency to avoid adverse effects on the temporomandibular joint. The unsuitable positioning of condyles represents one of the causative mechanism that may induce temporomandibular joint noxious effects among many factors [3]. The relationship between intraoperative malpositioning of the condyle and the occurrence of condylar resorption with relapse resulting in high levels of strain is well-known. Condylar resorption is a complication with a reported frequency of between 1.4% and 31% [3,4]. Relapse of the occlusal result can occur because of unduly posterior positioning of the condyle during mandibular advancement [5,6] or a high degree of rotation of the condyle during mandibular setback [7]. Plastic and maxillofacial surgery supervisors have an obligation to provide a training environment that includes evaluation mechanisms to certify acceptable acquisition of surgical skills [8,9]. Trainee learning curves for condylar positioning during BSSO are difficult to assess, especially using a manual empirical method. Traditional “observations-imitation” models based on the action of a supervisor require repeated practice and have steep learning curves. Dissection training is constrained and cannot offer condylar repositioning training because it may introduce damage to tissues which influences their anatomical position [10]. Despite of none scientific evidence supported the routine use of condyle positioning devices to prevent TMJ disorders [11–14], computer-assisted navigation could be considered as an educational tool to control the learning curve for condylar positioning among condylar positioning devices available to help surgeons in condylar repositioning [12]. Bettega et al. [15,16] have developed a condylar positioning system (CPS) based on navigation and underlined the benefits of using CPS routinely for clinical and educational purposes. The aim of this study was to evaluate the relevance of this computer-assisted surgery in acquiring condylar positioning competence.

## Materials and methods

**Fig 1:**
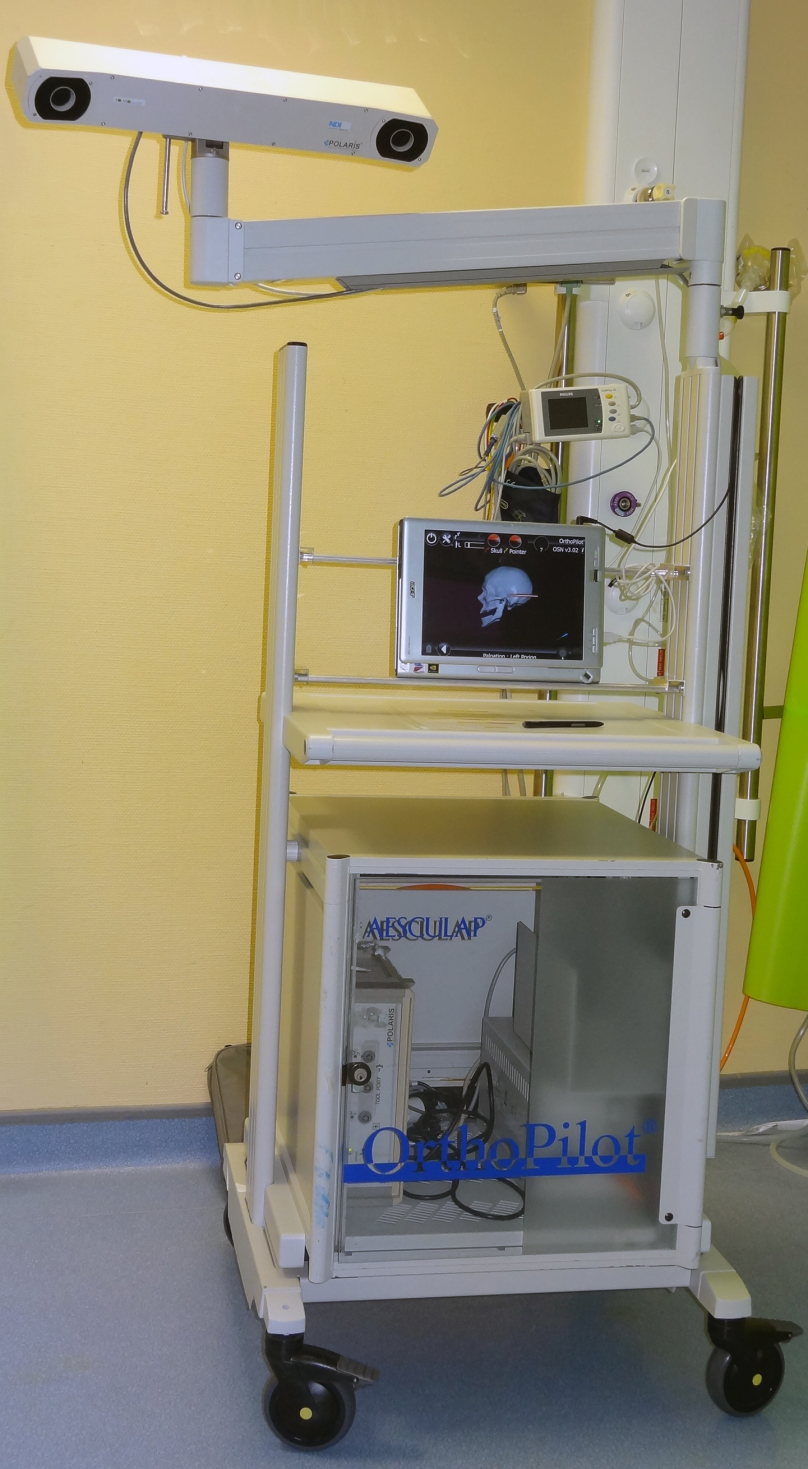
System with a 3D optical localizer including a double camera.

**Fig 2:**
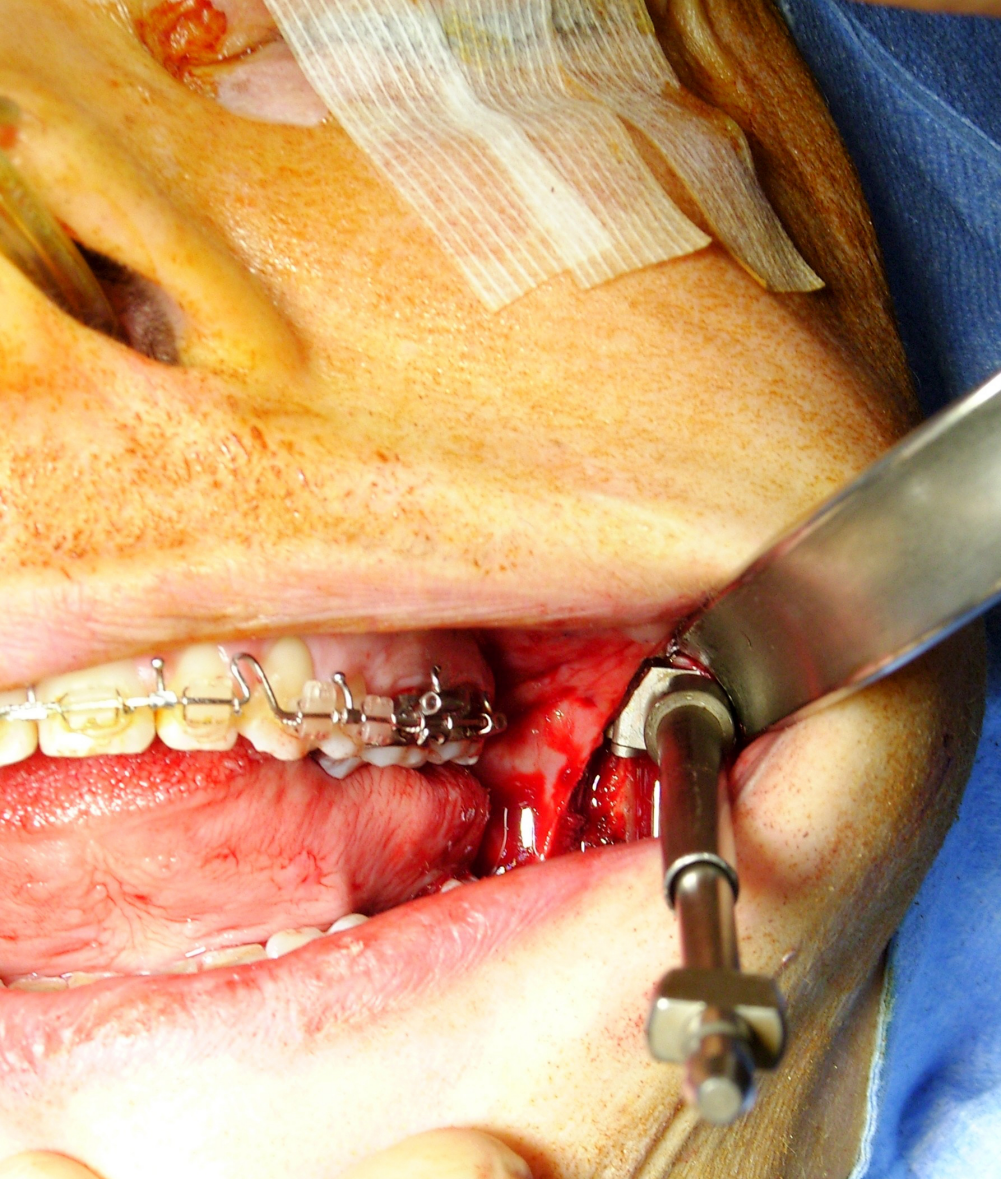
Infrared reflectors fixed on the coronoid process.

**Fig 3:**
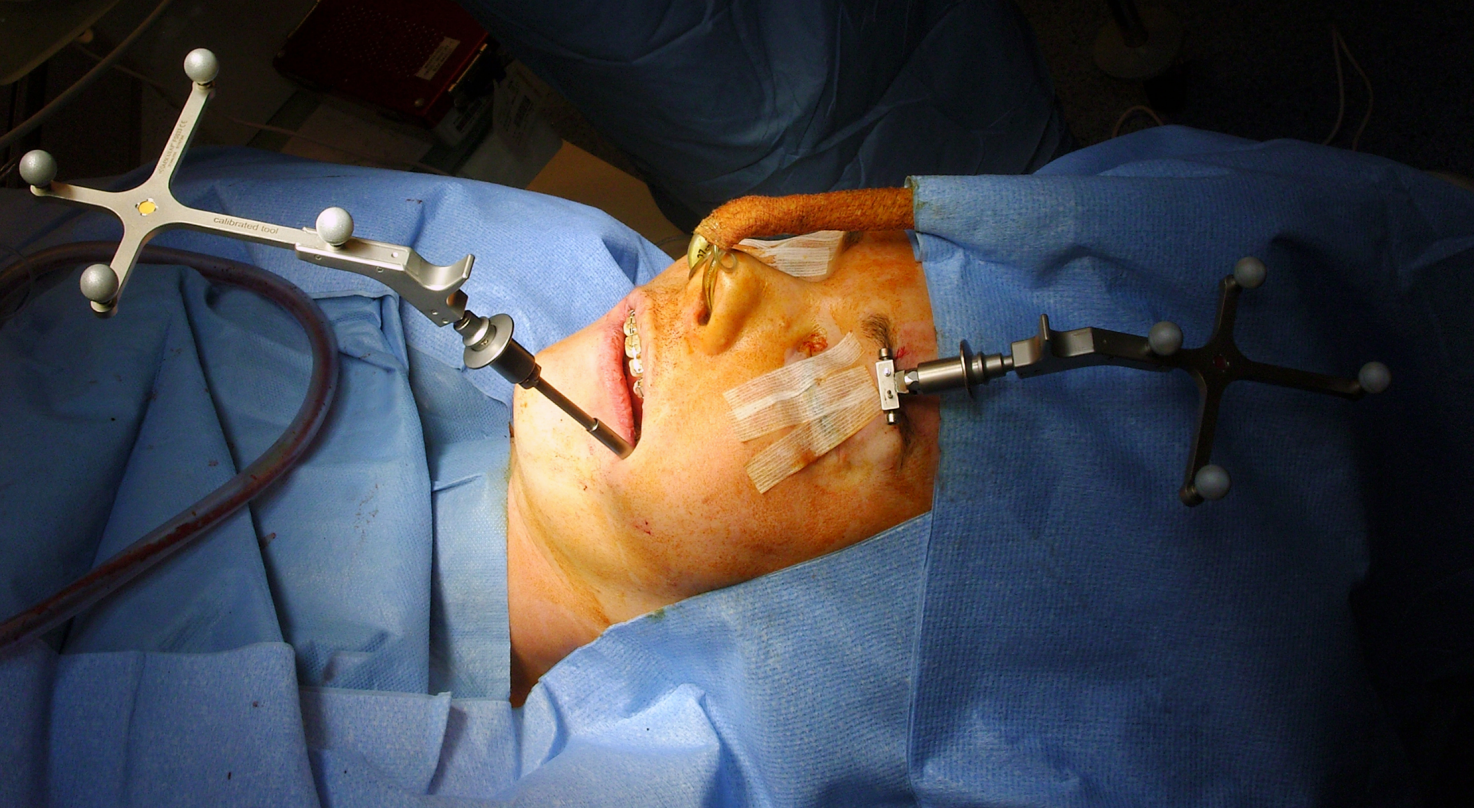
Infrared reflectors fixed on the orbital rim.

Forty-eight patients without temporomandibular joint disorder (TMD) underwent BSSO with osteosynthesis performed by six maxillofacial trainees. Two senior residents (trainees 1 and 6) had already undergone a period of supervised training with an experienced surgeon and the remaining four junior trainees had no experience (trainees 2, 3, 4, and 5). Patients with non-syndromic dentofacial deformity requiring isolated BSSO with or without bimaxillary osteotomy were included for analysis. One ramus was assessed regardless of the side and the opposite side was handled by an instructor. The condyle positioning system (CPS) developed by Bettega et al. [15,16] (and improved with technological progress) was used by a senior surgeon to record a condylar position score during the procedure. The system consisted of a 3D optical localizer, Orthopilot® (B. Braun-Aesculap, Tuttlingen, Germany), including a double camera (Polaris; NDI, Waterloo, Canada) (Fig 1) and infrared reflectors fixed on the coronoid process (Fig 2) and orbital ridge (Fig 3). The device fixed on the coronoid process was equipped with a connection that could hold a removable intermediary part to support infrared reflectors. Surgery began with the recording of the condyle reference position and identification of predetermined anatomic landmarks. The reference position corresponded to the centric relation that was established during the preoperative assessment and could be reproduced using a sterile centric relation splint. The patient’s centric relation bite was recorded using Dawson’s bilateral manipulation method [17] the day before the surgery in order to obtain the most posterior and superior location of the condyle in glenoid fossa. Reflectors fixed on the coronoid process were then removed and only the osseous anchorage remained, which completely freed the operation field.

**Fig 4:**
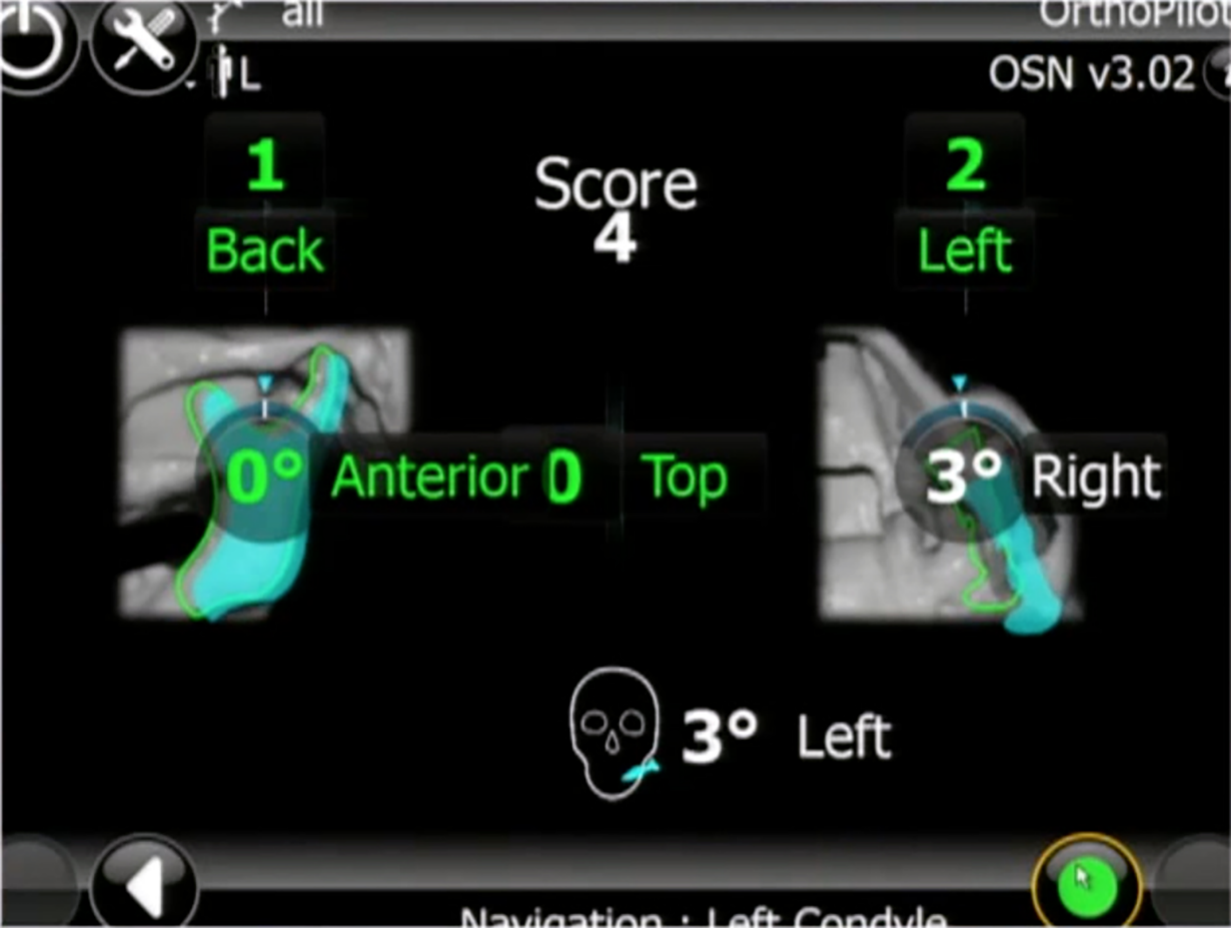
Tracking of condylar displacement (rotational and translational motions) on the computer screen. green: condylar fixed reference position, blue: mobilized bone segment by trainee (trainee position).

**Fig 5:**
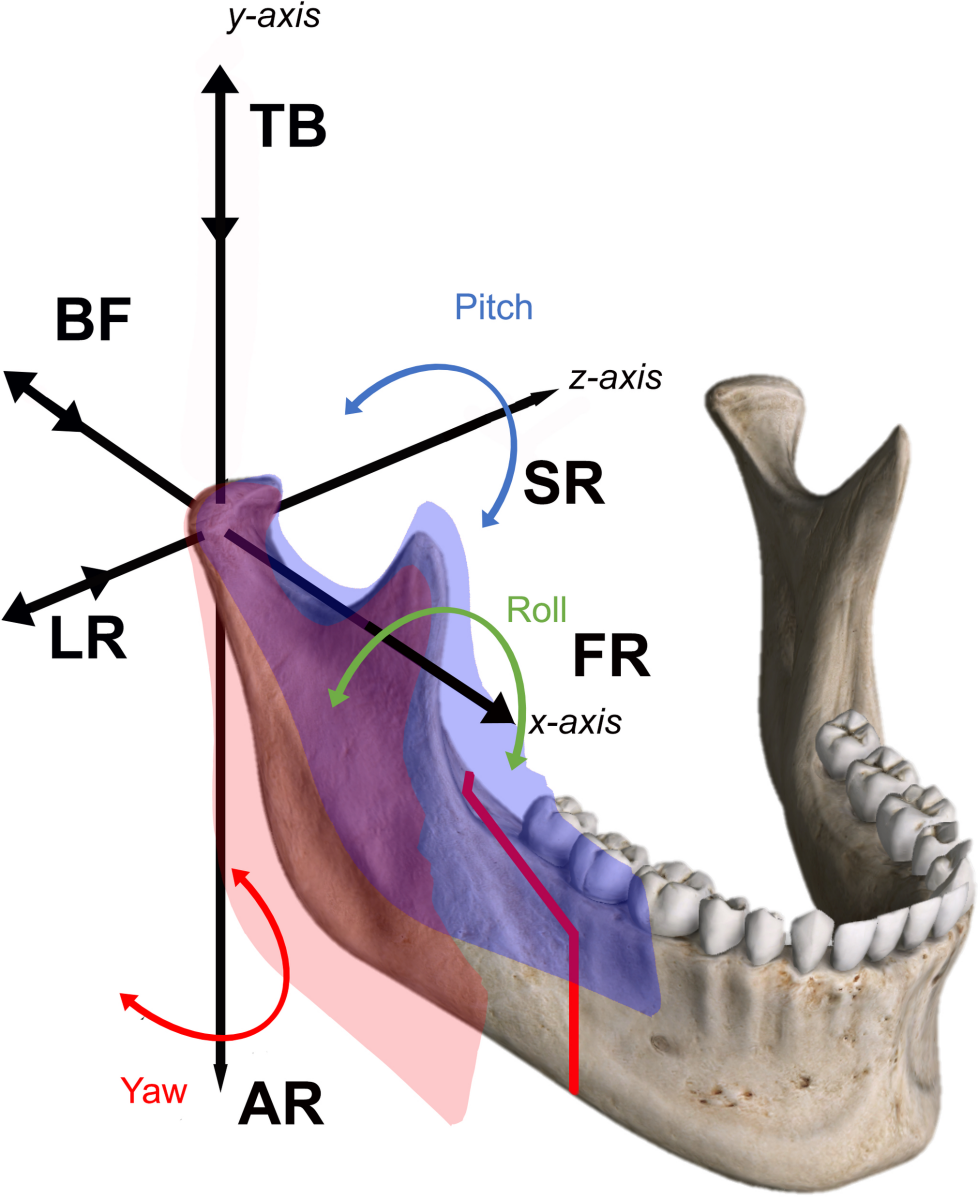
Translational and rotational displacements. Top to bottom (TB), back to front (BF), left to right (LR) and axial rotation (AR), sagittal rotation (SR), frontal rotation (FR), and total score (TS).

Each BSSO procedure was performed according to Obwegeser-Dal Pont [18,19]. Maxillomandibular fixation was then secured and the reflectors were reinserted on their respective anchorage to permit positional condyle assessment. The system provided control of the condylar fragment in a three-dimensional position and tracking of condylar displacement on the computer screen (Fig 4) during attempts to overlap the reference position (multimedia file). First, data were recorded by the supervisor as the trainee manually positioned the proximal segment without access to the CPS score (phase 1). Then, when the trainee positioned the proximal segment and performed osteosynthesis, they were given visual access to the CPS score (phase 2). The condyle position records during phase 2 were obtain before the osteosynthesis and then the osteosynthesis were performed in keeping the control of the condylar position on the screen. Osteosynthesis was performed by fixing three bicortical positional bone screws (2 mm diameter) placed using a transbuccal approach (S1 Video). Each trainee performed eight consecutive procedures. Six parameters describing condylar three-dimensional motions were assessed in phase 1 and phase 2 (Fig 5). First, the translational displacements were assessed: from top to bottom (TB), back to front (BF), and left to right (LR). Second, rotational displacements were assessed: axial rotation (AR), sagittal rotation (SR), frontal rotation (FR), and total score (TS), which was the sum of the scores in absolute values (TS=|TB|+|BF|+|LR|+|AR|+|SR|+|FR|). Translational and rotational parameters were defined in millimeters and degrees, respectively. Positive and negative signs indicated the direction of displacement. Quantitative variables were expressed as means and standard deviations (SD).

Qualitative variables were expressed as counts and percentages. Differences between groups were evaluated using the chi-square test or Fisher’s exact test for categorical variables and the t test for continuous variables. Linear mixed effect models were used to analyze the evolution of the measured value with increases in the number of interventions. Statistical analyses were performed using R.3.2.3 software.

A positive IRB n°5921 (Ethic committee, CECIC Rhône-Alpes-Auvergne) expeditive review was obtained (S1 Text) and all patients were informed orally of the protocol with writing consent, risks and use of the data for biomedical research.

## Results

The mean age of patients who underwent BSSO was 21.98 years, and 27 (56.25%) were female and 21 (43.75%) were male. Twenty-four patients received an isolated mandibular osteotomy (± genioplasty) and the other 24 cases underwent bimaxillary procedures (± genioplasty) (Table 1). One ramus for each patient was used to assess the condylar positioning of trainees. No specific complications related to the use of the system were noted. There was no significant difference in the positioning of condyles for the junior and senior residents in phase 1 and phase 2 (Table 2). Condyles were positioned with better control during phase 2 using the CPS for all trainees. The mean (SD) of absolute values was 2.56 (2.36) during phase 1 and 1.37 (1.25) during phase 2 (*p*<0.001) (Table 3). Results showed that rotational movements in the axial axis (AR) with a mean of 4° (*p*<0.001) and translational movements in the left to right (LR) dimension with a mean of 2.48 mm (*p* = 0.013) were more difficult and less accurate to handle during phase 1 for all trainees (Table 3).

**Table 1:** Summary of the population and the surgical procedures.

Patient (N°)	Gender	Age (year)	Indication	Surgical procedure	Trainee-Intervention (n°-n°)	Side
**1**	M	22	Mandibular Class II	BSSO advancement of 6 mm, genioplasty	1–1	L
**2**	F	16	Mandibular Class II	BSSO advancement of 9 mm	1–2	L
**3**	F	20	Open bite Class III	Le Fort I advancement, BSSO asymmetric set back 2 mm	1–3	L
**4**	M	24	Open bite Class II	Le Fort I advancement, BSSO advancement 9 mm, genioplasty	2–1	R
**5**	F	21	Class III	Le Fort I advancement, BSSO setback 3 mm	1–4	L
**6**	F	17	Mandibular Class II	BSSO advancement of 7 mm	2–2	L
**7**	F	15	Mandibular Class II	BSSO advancement of 5 mm	1–5	R
**8**	F	16	Mandibular Class II	Le Fort I advancement, BSSO asymmetric advancement 4 mm	2–3	R
**9**	F	22	Mandibular Class II	BSSO advancement of 10 mm	2–4	R
**10**	M	22	Open bite Class II	Le Fort I advancement, BSSO advancement 10 mm, genioplasty	2–5	L
**11**	M	24	Mandibular Class II	Le Fort I advancement, BSSO asymmetric advancement 7 mm, genioplasty	1–6	L
**12**	M	16	Open bite Class III	Le Fort I advancement, BSSO asymmetric set back 2 mm	2–6	L
**13**	F	16	Mandibular Class II	BSSO advancement of 7 mm	1–7	R
**14**	M	18	Open bite Class II	Le Fort I advancement, BSSO advancement 8 mm	1–8	L
**15**	M	22	Mandibular Class II	Le Fort I reposition, BSSO asymmetric advancement 4 mm, genioplasty	2–7	R
**16**	M	19	Mandibular Class II	BSSO asymmetric advancement of 7 mm	2–8	L
**17**	F	17	Mandibular Class II	BSSO advancement of 7 mm, genioplasty	3–1	R
**18**	M	21	Mandibular Class II	BSSO advancement of 6 mm	3–2	R
**19**	M	37	Mandibular Class II	BSSO advancement of 6 mm, genioplasty	3–3	R
**20**	F	16	Mandibular Class II	BSSO advancement of 9 mm, genioplasty	3–4	L
**21**	M	16	Class III	Le Fort I advancement, BSSO set back 3 mm	3–5	L
**22**	M	23	Class III	Le Fort I advancement, BSSO set back 3 mm, genioplasty	3–6	L
**23**	F	21	Open bite Class III	Le Fort I advancement, BSSO asymmetric set back 3 mm	3–7	L
**24**	M	18	Mandibular Class II	Le Fort I reposition, BSSO advancement of 10 mm	3–8	L
**25**	F	19	Asymmetrical Class II	Le Fort I frontal rotation for midline correction, BSSO asymmetrical advancement 9 mm	4–1	L
**26**	M	24	Mandibular Class II	BSSO advancement of 9 mm	4–2	L
**27**	F	20	Asymmetric Class I	Le Fort I frontal rotation for midline correction, BSSO	4–3	L
**28**	M	17	Open bite Class II	Le Fort I advancement, BSSO advancement 9 mm, genioplasty	5–1	R
**29**	M	17	Mandibular Class II	BSSO advancement of 8 mm	6–1	L
**30**	F	16	Mandibular Class II	BSSO advancement of 9 mm	5–2	L
**31**	F	56	OSAS	Le Fort I + BSSO advancement of 12 mm	5–3	L
**32**	F	19	Open bite Class III	Le Fort I advancement, BSSO asymmetric set back 2 mm	4–4	L
**33**	F	20	Mandibular Class II	BSSO advancement of 8 mm	6–2	R
**34**	F	24	Mandibular Class II	BSSO advancement of 7 mm	4–5	L
**35**	M	16	Mandibular Class II	BSSO advancement of 7 mm	4–6	R
**36**	F	32	Open bite Class II	Le Fort I advancement, BSSO advancement 11 mm, genioplasty	4–7	R
**37**	M	29	Mandibular Class II	BSSO advancement of 6 mm	5–4	R
**38**	F	30	Open bite Class II	Le Fort I advancement, BSSO advancement 14 mm, genioplasty	4–8	R
**39**	M	17	Mandibular Class II	BSSO advancement of 6 mm	5–5	L
**40**	M	20	Mandibular Class II	BSSO advancement of 5 mm, genioplasty	6–3	R
**41**	F	22	Class III	Le Fort I advancement, BSSO set back 3 mm	5–6	R
**42**	F	30	Open bite Class II	Le Fort I advancement, BSSO advancement 9 mm, genioplasty	5–7	L
**43**	F	24	Mandibular Class II	BSSO asymmetric advancement of 7 mm	6–4	L
**44**	F	42	OSAS	Le Fort I + BSSO advancement of 12 mm	5–8	L
**45**	F	16	Mandibular Class II	BSSO advancement of 9 mm	6–5	R
**46**	M	18	Open bite Class III	Le Fort I advancement, BSSO asymmetric set back 2 mm	6–6	L
**47**	F	25	Mandibular Class II	BSSO advancement of 6 mm	6–7	R
**48**	F	23	Mandibular Class II	BSSO advancement of 10 mm, genioplasty	6–8	R

M, male; F, female; L, left; R, right; BSSO, bilateral sagittal split osteotomy; OSAS, obstructive sleep apnea syndrome.

**Table 2:** Difference in the positioning of condyle between the junior and senior residents in phase 1 and phase 2.

	Phase 1			Phase 2		
	Junior trainees Score (SD)	Senior trainees Score (SD)	*p* (t-test)	Junior trainees Score (SD)	Senior trainees Score (SD)	*p* (t-test)
**TB (mm)**	1.95(1.61)	1.7(1.16)	0.652	1.16(0.95)	1.1(0.88)	0.862
**BF (mm)**	1.08(1.08)	1.5(1.18)	0.285	0.97(0.75)	1.1(0.74)	0.638
**LR (mm)**	2.45(2.04)	2.6(1.71)	0.829	1.47(1.11)	2.3(2.11)	0.095
**FR (°)**	2.24(2.12)	3(1.94)	0.31	1.61(1.22)	2.7(2.36)	0.047
**AR (°)**	4.26(2.8)	3(2.05)	0.189	1.68(1.68)	1.2(0.63)	0.378
**SR (°)**	3.74(3.25)	2.1(2.23)	0.142	1.13(0.88)	0.6(0.7)	0.083
**TS**	15.71(7.01)	13.9(4.41)	0.132	8.03(3.28)	9(3.13)	0.403

TB, top to bottom translation; BF, back to front translation; LR, left to right translation; FR, frontal rotation; AR, axial rotation; SR, sagittal rotation; TS, total score; mm, millimeters; °, degrees; SD, standard deviation. Scores in absolute values.

**Table 3:** Difference in the positioning of condyle between phase 1 and phase 2 for all trainees.

	Junior and senior trainees		
	Phase 1 Score (SD)	Phase 2 Score (SD)	*p* (student test)
**TB (mm)**	1.9 (1.52)	1.15 (0.92)	0.003
**BF (mm)**	1.17 (1.1)	1 (0.74)	0.364
**LR (mm)**	2.48 (1.96)	1.65 (1.39)	0.013
**FR (°)**	2.4 (2.09)	1.83 (1.56)	0.104
**AR (°)**	4 (2.69)	1.58 (1.53)	<0.001
**SR (°)**	3.4 (3.12)	1.02 (0.86)	<0.001
**TS**	15 (6.55)	8.23 (3.24)	<0.001
**Mean**	2.56 (2.36)	1.37 (1.25)	<0.001

TB, top to bottom translation; BF, back to front translation; LR, left to right translation; FR, frontal rotation; AR, axial rotation; SR, sagittal rotation; TS, total score; mm, millimeters; °, degrees; SD, standard deviation. Score (mean of absolute values).

**Fig 6:**
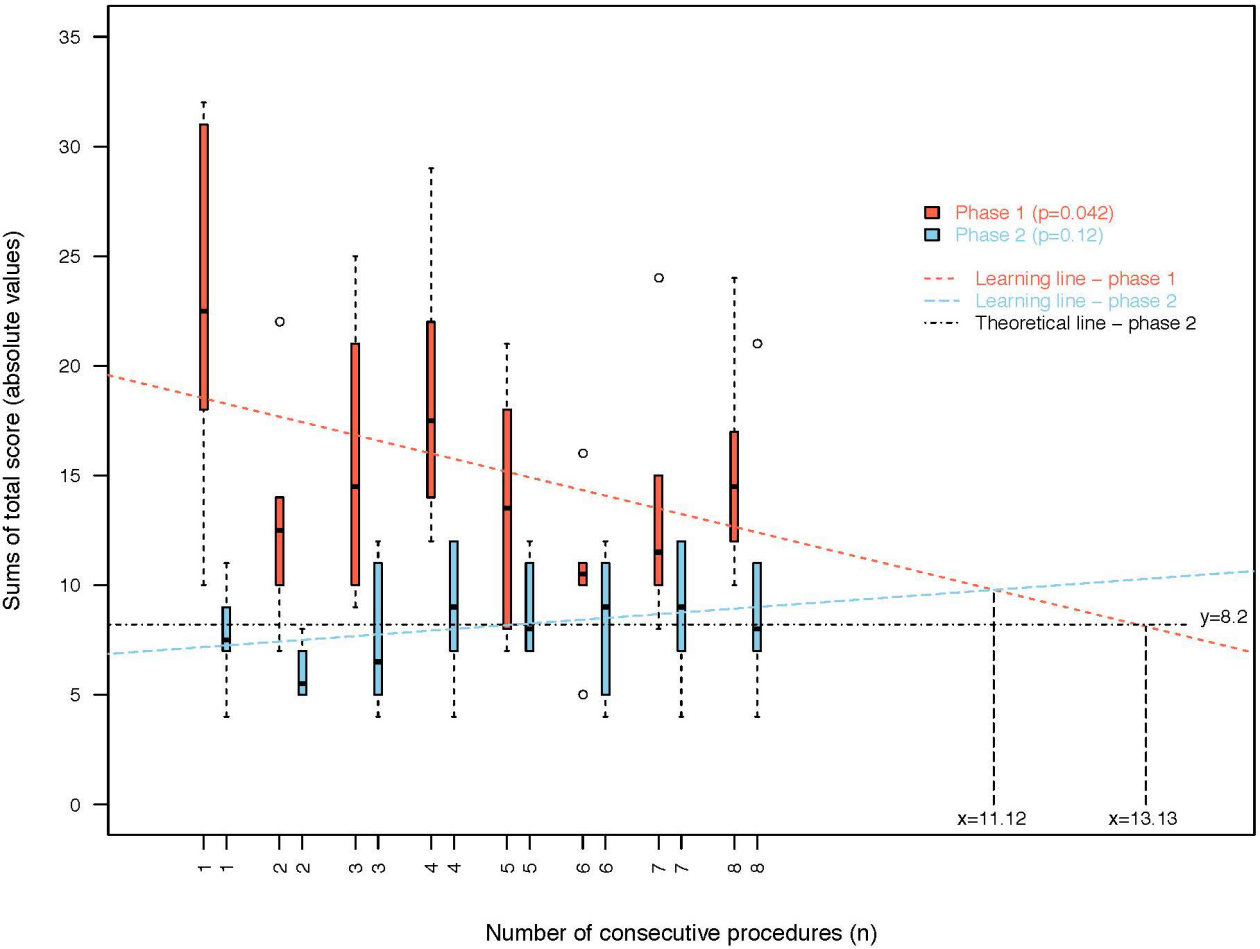
Total scores (TS) evolution according to the number of procedures.

**Fig 7:**
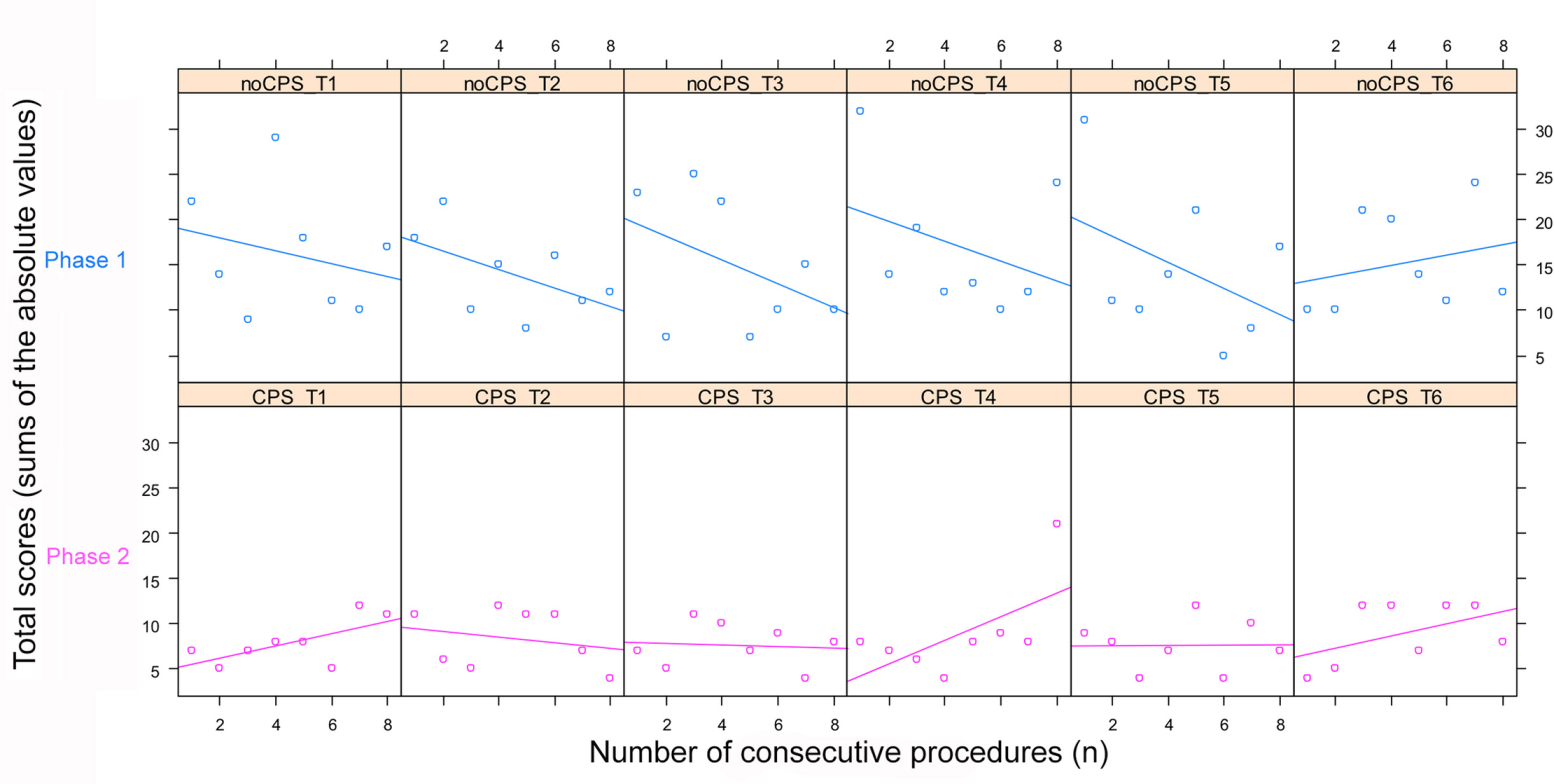
Total score (TS) of each trainee with consecutive procedures. CPS: condyle positioning system.

Over eight consecutive procedures, significant global improvements in total scores (TS) (sums of the absolute values) were seen in phase 1 (*p* = 0.042) for trainees (Fig 6), and there was a non-significant deterioration in scores in phase 2 (*p* = 0.12). Fig 7 shows the total score (TS) of each trainee, and only results for trainee 6 did not improve in phase 1.

The evolution of the score lines for phase 1 and phase 2 showed a crossing after 11 procedures. The theoretical line of no-deterioration in phase 2 should have crossed the phase 1 line after 13 procedures (Fig 6).

## Discussion

Most maxillofacial surgeons use an intuitive empirical method for repositioning the condylar fragment during BSSO [2,12]. The condyle centric position used as a reference is not a physiological position but rather a border position that is used for reproducibility. Surgeons rely on manual repositioning after BSSO in order to obtain the best mandibular proximal segment relationship with the condylar fossa [13]. Acquisition of this “intuitive” technical skill using the traditional ‘‘apprentice–mentor” education model produces a lengthy learning curve in which performance tends to improve after a long period of experience [20,21]. Acquisition of condylar repositioning skills mastered using observation, followed by imitation of the mentor’s actions can be difficult for trainees and this can result in adverse functional implications following this technically demanding step. Condyle repositioning devices are usually used by clinicians who do not wish to intuitively reposition the condylar fragment during BSSO [12]. Among many condyle repositioning techniques, the navigation technique developed by Bettega et al. [15,16] can improve condyle position within a precision of 1 mm in translational movements and 1° in rotational movements. Elfring et al. [22] assessed the accuracy of the optical localizer system used in our study and showed equal precision.

Bettega et al. [15,16] compared groups of patients which underwent BSSO with standard technique (empirical group) and BSSO with CPS (active group) and assessed postoperative occlusion, stability of skeletal position, the preservation of mandibular motion and the occurrence of temporomandibular dysfunction with 12 months follow up. Forty five percent of empirical group showed worsened temporomandibular joint status and only one in the active group. Despite of the previous results we do not overestimate the usefulness of CPS because our study did not provide any results on the occurrence of the temporomandibular joint disorders and we emphasize the educational interest of CPD more than its clinical importance.

Computer-aided innovations have become routine practice in oral and maxillofacial surgery teaching [23,24]. To the best of our knowledge, no study has evaluated the contribution of navigation devices for skill development. In a recent review, Azermehr et al. [25] described the most common indications for navigation devices in all fields of oral and maxillofacial surgery; however, this has not raised interest. Therefore, the aim of this study was to evaluate the relevance of a CPS device that provides graphical results of performance against experience as a learning tool to facilitate a short learning curve.

In our orthognathic center, we use navigation systems and computer-aided BSSO surgery to help surgeons in replacing the condylar process, and therefore, the implementation of this device for our research was simple to establish. This study assessed clinical practice and was not a clinical research study. The navigational device used in this study removed the need for preoperative CT imaging, and therefore, unnecessary patient irradiation that occurs with other navigational techniques [26]. Furthermore, it prevents registration of data problems by the computer algorithm, which could affect the precision and accuracy of the navigation system [27].

**Fig 8:**
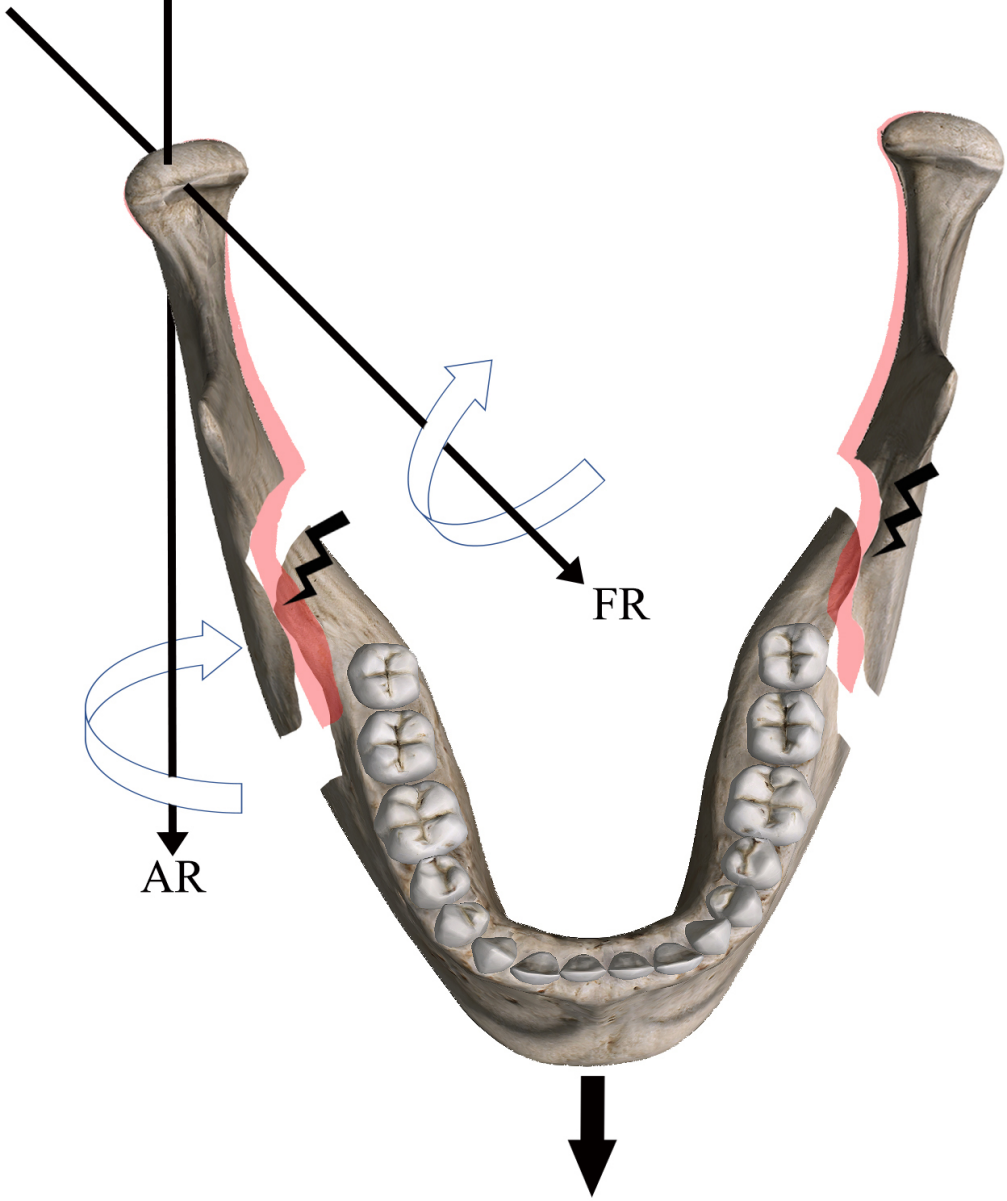
Interference of posterocaudal bony segments with axial and frontal condylar segment rotation after excessive mandibular advancement.

Our study showed a significant improvement in condyle placement over consecutive procedures with a crossing of the phase 1 and phase 2 lines after 10 procedures. Theoretically, the phase 2 line should have been “flat” without any increase or decrease in slope but there was a non-significant deterioration in the overall score. This was due to the bad replacement performed in patient n° 38 by trainee 4 during the last procedure (Table 1), with a total score of 24 in phase 1 and 21 in phase 2 (Fig 7). This procedure was a crucial mandibular advancement resulting in an internal segment and proximal condylar segment conflict [28]. This might have led to the interference of unidentified posterocaudal bony segments with the final positioning of the condylar segment, showing that placement in a similar preoperative position is still challenging after excessive mandibular movement [29] (Fig 8). Indeed, it is commonly accepted that advancements greater than 10 mm expose condyles to condylar resorption due to an increasing of tension of the surrounding soft tissues producing an inferior-posteriorly directed force [3,30]. Condyle rotation induces excessive mechanical stress and the adaptive capacities of the host are diminished, the remodeling becomes dysfunctional, and this leads to condylar resorption. This demonstrates, once again, to the importance of leaving the condyle in a position as close as possible to the preoperative position. Trainee 4 could not correctly relocate the condyle axial rotation movement, which is often the case in large advancements. Therefore, we drew a theoretical line of optimal placement of the condyle. The phase 1 line crossed this theoretical line after about 13 procedures. We can assume that regardless of the type of BSSO, the learning curve plateau after 13 procedures, which is supposedly a shorter period compared to traditional learning. On the other hand, the experienced trainee 6 was the only trainee to not improve during phase 1, perhaps due to prior experience in condylar repositioning.

To the best of our knowledge, no study has assessed the learning curves for condylar positioning in trainees to determine the number required procedures without (and with) CPS. Future studies should assess the effectiveness of non-tutored practice of condylar positioning during phase 1 of BSSO to clarify this point. The highly individual learning curves suggest a predefined number of practice sessions before a mean plateau is reached, highlighting the maximum possible benefit. However, achieving this level is dependent on defining such a standard first, and ideally using a validated performance assessment tool. Most reported instruments for condylar segment repositioning performance assessment, such as cephalometric analysis [31], CT [5], cone beam CT (CBCT) [32,33], and finite element analysis [34], can capture position aspects, but only in post-operative conditions. The CPS appears to be an efficient intra-operative learning tool that is completely safe. Nonetheless, all of these methods are dependent on the time-consuming rating of performance using trained experts’ context of deliberate practice and training towards a standard set of proficiency levels.

Our study assessed the learning curve of condyle repositioning regardless of the side of the BSSO. However, it seems essential to pay attention to the effect of the dominant hand and side on the ability and confidence in using powered instruments. Abdel-Galil et al. [35] assessed trainers and trainees during BSSO, and confirmed that most operators were right-handed and most trainees surveyed reported that they operated predominantly from the left side when doing a BSSO, whereas their trainers operated predominantly from the right side. It is our practice that trainers and trainees alternate operating sides for consecutive cases, allowing the trainee to experience performing the same procedure using different approaches, as well as learning the motor skills required to use the non-dominant hand, thereby improving confidence and safety.

The length of time taken for the procedure was not included as a study parameter and the “learning curve” was also defined as improving performance over time or increasing experience or training. In retrospect, we should have undertaken a “learning curve cumulative sum analysis (LC-CUSUM)”, which is a sequential analysis tool originally developed for quality control purposes according the duration of the procedure and can be used to judge when an individual’s performance has reached a predefined level of competence [36]. This statistical tool would allow evaluation of the specific influence of the CPS in the learning of repositioning skills during phase 1 after consecutive procedures, and not only in phase 2.

Learning and skills acquisition is dependent on memory consolidation, and the spacing of practice allows this to occur [37]. The optimal intertraining interval for skills practice and consolidation (distributed practice) is still debated in the literature, but has been demonstrated to benefit complex psychomotor skills acquisition in virtual reality simulated laparoscopy training [38]. Our study was based on consecutive practice and did not assess the effects of intertraining intervals which ranged from 1 day to many days. However, this parameter is more suitable for the learning of virtual reality simulation surgery [37]. The optimal intertraining interval for skills practice and consolidation is still debated in the literature [39]. Learning and skills acquisition is dependent on memory consolidation, and spacing of practice allows this to occur [40]. A minimal of three intertraining days is considered as sufficient for improving novice performance [40]. In our study this intertraining delay was respected.

Various virtual reality simulators and haptic devices have been proposed and implemented in past years [23,41]. Surgical simulation offers a near-realistic training environment, with the possibility for unlimited training over a wide variety of procedures. However, simulation is not suitable for condylar positioning skill development as the simulation of realistic force feedback is not possible. Furthermore, positioning guides and pre-bended osteosynthesis plates are being increasingly used in orthognathic surgery owing to their accuracy. These guides are manufactured using computer-aided design/computer-aided manufacturing (CAD/CAM) technologies after digital surgery planning rather than conventional planning to provide better accuracy [42][43]. Condyle positioning is predetermined during the digital planning and this offers facilitation of better intraoperative condyle positioning, but this cannot be considered as a learning tool.

In our study, all BSSO osteosynthesis procedures involved rigid fixation and the fixation of three bicortical positional bone screws. Rigid and non-rigid fixation in BSSO are important considerations in the condylar segment positioning learning curve. Indeed, the technical management is different for the two procedures, and clinicians are concerned that rigid internal fixation can induce great changes in the position of the condyle compared to non-rigid fixation [33,44,45]. The use of condylar positioning devices seems reasonable for practice and learning [12], but there are still controversies. In a future study, we will assess the condylar positioning with non-rigid fixation learning curve using the same procedures and will compare results to the results of this study.

We have emphasized in this study the importance of the condyle position during BSSO but we highlight the influence of the closing and opening muscle groups of the jaw on the stress distribution in the condyles with changing jaw position after BSSO with condylar resorption consequence [34,46–48].

We must remember that the condylar positioning step of BSSO is critical to learn, but the acquisition of osteotomy skills is also crucial and cannot be assessed with the CPS. The development of a simulator that provides training for all stages of BSSO would be ideal.

## Conclusion

Our results show that condylar repositioning skill development based on navigational computer-assisted surgery is effective for trainees, and the acquired skills are qualitatively and quantitatively measurable. CPS provides for trainees to obtain a condyle position reference positioning during bilateral sagittal split osteotomy and limits the excessive movement of the condyles. The use of a CPS is an effective aid in condylar repositioning and can be consider as a tool for acquiring condylar positioning skills, particularly taking into account the reduced working hours and increased number of residents in hospitals, which may result in less exposure to surgery. Our study showed only this educational interest, however further studies need to carry out to assess the relationship between the use of CPS and less occurring of temporomandibular joint disorders.

Constant innovations in treatment modalities means there are always new surgical procedures to be learnt by supervisors and mentors and this reduces the number of surgical procedures available for the teaching and learning of apprentices. Furthermore, the continuous pressure to reduce operation times in order to be more cost effective, and the ethical aspects to limit patient morbidity, reduce complications, and maximize patient safety drive public awareness and demand professional responsibility.

## Supporting information

S1 Video Study methodology. (MP4)

S1 Text Ethic statement. (PDF)
