# Supplementary material for: Computer-assisted teaching of bilateral sagittal split osteotomy: Learning curve for condylar positioning
Source: PLoS One. 2018 Apr 25;13(4):e0196136. doi: 10.1371/journal.pone.0196136 (PMC5918964; doi:10.1371/journal.pone.0196136)
Supplement: S1 Text — (PDF) [file pone.0196136.s002.pdf]

Comité d'Ethique des Centres d'Investigation Clinique de l'inter-région Rhône-Alpes-Auvergne

Dr Christian Dualé
Chair
Centre de Clermont-Ferrand
IRB n°00005891
cduale@chu-clermontferrand.fr
Tél. 04.73.17.84.18
Fax 04.73.17.84.12

Pr Jean-Luc Cracowski
Chair
Centre de Grenoble
IRB n°00005921
cic@chu-grenoble.fr
Tél. 04 76 76 92 60
Fax 04 76 76 92 62

Evaluation éthique d'une recherche par le CECIC Rhône-Alpes-Auvergne

February 13th, 2018

Dear Pr Bettega,

Following our discussion and an expeditive review of your study, I confirm you that in France, there is no formal need that your project undergoes a formal review of your study entitled "Computer-assisted teaching of bilateral sagittal split osteotomy: learning curve for condylar positioning." by Charles Savoldelli, Emmanuel Chamorey, Georges Bettega.

In my opinion, this study follows the standards of care, and is a retrospective analysis of data acquired in a hospital setting. Therefore, in an expeditive review process, as the chair of the comité d'éthique des Centres d'investigation clinique de l'inter-région rhône-alpes –Auvergne, registered as IRB n°5921 on http://ohrp.cit.nih.gov/ , I can confirm that your project meets the ethical standards of our IRB and is given a positive IRB review. Should the journal editor need it, he may contact me directly.

Should you wish to have a full review of your project, this would not be possible within 4 weeks, given that we unfortunately currently have delays for the full review process.

Jean-Luc Cracowski, chair
